# The importance of neurosurgical nursing in low- and middle-income countries – a critical review

**DOI:** 10.1097/MS9.0000000000002910

**Published:** 2025-01-31

**Authors:** Samuel Inshutiyimana, Olivier Uwishema, Nagham Ramadan, Laura Ghanem, Zeina Al Maaz, Victoire Mukamitari, Magda Wojtara, Sarah Mshaymesh

**Affiliations:** a Department of Research and Education, Oli Health Magazine Organization, Kigali, Rwanda; b Department of Pharmaceutics and Pharmacy Practice, School of Pharmacy and Health Sciences, United States International University-Africa, Nairobi, Kenya; c Department of Medicine, Faculty of Medicine, Beirut Arab University, Beirut, Lebanon; d Faculty of Medical Sciences, Lebanese University, Beirut, Lebanon; e Department of Surgery, College of Medicine and Health Sciences, University of Rwanda, Kigali, Rwanda; f Department of Human Genetics, University of Michigan Medical School, Ann Arbor Michigan; g Department of Natural Sciences, Faculty of Sciences, Haigazian University, Beirut, Lebanon

**Keywords:** LMICs, neurosurgery, neurosurgical care, neurosurgical nurses, neurosurgical nursing

## Abstract

**Background:**

Neurosurgical nursing involves the provision of pre- and post-operative care to neurologic patients. Specialized nurses in the field of neurosurgery are vital for patient outcomes and wellbeing. Nevertheless, there is underestimation and limited access to neurosurgical nursing in low- and middle-income countries (LMICs). This review primarily aims to shed light on the significance of nursing in the neurosurgical department of LMICs. It provides insight into the healthcare systems, the collaborative approaches that emerged in this concern, and the issues pertaining to integrating neurosurgical nursing in these countries.

**Methods:**

Literature search was conducted from March 2024, where the articles were retrieved from PubMed/Medline, EBSCOhost, Google Scholar, and Embase. The terms “neurosurgical nursing,” “neurosurgery,” “neurosurgical care,” “neurosurgical nurses,” and “low- and middle-income countries OR LMICs” were used to search relevant studies. Previous articles discussing neurosurgical care in LMICs were considered in this review.

**Results:**

Nurses participate in 90% of interactions between healthcare providers and patients. They provide holistic care by considering the physical, cultural, and psychosociological needs pertaining to their patients. Furthermore, they possess ability to establish rapport via communication with patients which improves the standard of care delivered. To achieve results satisfactory to the wellbeing of affected populations, neurosurgical disciplines necessitate the aid of skilled nursing colleagues. However, there is a lack of relevant technology, innovations, and funding alongside human resources in LMICs.

**Conclusion:**

Education, training, and dynamic collaboration are crucial factors for neurosurgical nurses to provide the best quality of care to patients. It is also pivotal to focus on research concerning the present issue, implement further policies which encourage dynamic cooperation between the neurosurgical multidisciplinary team, and collaborate on a global level to enhance neurosurgical nursing in LMICs.

## Introduction

The World Bank, in 2022, classified 65% of countries globally under the low- and middle-income category. This classification is based on gross national income (GNI) per capita of each country where the low-income countries have a GNI per capita of less than or equal to United States dollars (US $) 1135. Besides, lower middle-income countries have a GNI per capita that falls in the range of US $ 1136 to US $ 4465. However, a GNI per capita of $13 846 or more is demonstrated by high-income countries (HICs)^[1,2]^. This financial discrepancy between countries poses challenges to low- and middle-income countries (LMICs), especially in the healthcare sector. Such countries find it difficult to afford reliable medical equipment and supplies alongside the maintenance of sound clinical practices for proper hygiene and infection control to be. Furthermore, there is a shortage of healthcare support services within training, education, and human resource departments^[3,4]^. Studies have shown that healthcare workers in low-income countries decide to permanently leave jobs based in their respective country of origin to pursue their career in HICs which offer better minimum wage and more desirable training services^[5]^. Thus, LMICs struggle in securing adequate healthcare services in a wide array of medical disciplines, including neurosurgery^[4]^. In sub-Saharan Africa, 25.26% of patients have access to neurosurgical services within a 2-hour window compared to a substantial 93.3% in Eastern Europe and Central Asia^[4]^. Hence, the aim of this paper is to critically review the significance of nursing care in the neurosurgical department, the collaborative approaches that emerged in this concern, and the issues pertaining to integrating neurosurgical nursing in LMICs.

### Understanding neurosurgical nursing

Neurosurgical patient care, whether pre- or post-operation, is managed mainly by nurse practitioners in the field of neurosurgery. Neurosurgical nurses work with a wide spectrum of patients, from chronic neurodegenerative diseases like dementia to acute cerebrovascular and traumatic brain injury cases comprising stroke, contusion, and injury to the spinal cord. Neurosurgical nurses foster a major role in assessing the neurological status of patients while monitoring vital signs, anticipating any neurological sequalae such as post-operative increase of intracranial pressure due to hemorrhage, detecting early signs of infection, and pain management, among others^[6]^. Mostly employed in inpatient hospital settings, they focus efforts in educating patients and their families about procedures and results, providing detailed information alongside emotional support^[6]^. Studies have elucidated that specialized nurses in the field of neurosurgery are vital for patient outcomes and wellbeing. Patients who have been supervised by incompetent nurses in such specialties had poorer outcomes than those who were assigned a component neurosurgical nurse^[7]^. In addition, neurosurgical nurses are trained to be able to organize and maintain satisfactory surgical environments by ensuring all equipment provided beforehand, setting up the neurosurgical multidisciplinary team, maintaining professional communication inside the operating room, and being competent in case of any necessary unforeseen troubleshooting^[7,8]^. Moreover, neurosurgical nurses are required to facilitate patients triage especially during endemics of unprecedented etiology^[9]^. Therefore, it is imperative to install specialized training and skillsets to fulfill neurosurgical needs. Neurosurgical nurses, through their training, devotion to the surgical specialty, and educational attainment, are able to enhance patient safety and postoperative outcomes by recognizing and responding to any subtle changes that may occur to the status of their patients, thus preventing complication and enhancing recovery following surgery^[10]^. In addition, specialized training and knowledge allows neurosurgical nurses to contribute to informed decision making and effective care planning under a multidisciplinary team^[11]^. They can provide comprehensive care addressing patient physical, emotional, and psychosociological health^[12]^ (see Table 1).

**Table 1:** Key responsibilities and roles of neurosurgical nurses.

Key responsibilities and roles	Career traits	References
Preoperative assessment of safety checklists to assist in coordinating team and maintain safe environment	Competency in verification	^[7,13]^
Assess that all equipment are available and well-functioning	Team competency and good leadership	^[7,8]^
Post-operative patient supervision	Repetitive use of knowledge and skills needed in complex procedures	^[14]^
Coordination with radiological department to ensure imaging pre-operatively	Fully committed	^[8]^
Facilitate outpatient neurosurgical care	None	^[15]^
Advocating for patient’s needs and preferences within the team	None	^[6]^
Patient rehabilitation post operatively	None	^[10]^

## Discussion

### Healthcare landscape in LMICs

In LMICs, healthcare has been a longstanding subject of interest, encompassing institutions, organizations, and resources ranging from physical to financial. Many studies have shown the lack of stable healthcare systems in LMICs^[16]^. The shortage in financial support from different governments has led millions of their respective citizens to be placed under the poverty threshold therefore being unable to afford the luxury of medical attention^[17]^. Furthermore, the limited access to and implications of emerging technologies have been a limiting factor in the improvement of healthcare services in developing countries^[18]^. Most medical devices are developed in HICs and require adequate environmental and maintenance systems. As a result, developing countries have been relying on subpar methodologies to deliver such techniques^[19]^. Consequently, the delivery of adequate neurosurgical care has been challenging in LMICs. One significant issue that faces affected domiciles is the scarcity of professionally trained neurosurgeons to care for high-risk patients in very large populations, in addition to the very limited neurosurgical care^[20]^. Graduates of surgical programs in LMICs frequently relocate to other countries primarily for professional reasons, rather than for socioeconomic or personal motives. This migration is often driven by dissatisfaction with medical infrastructure, a shortage of surgical peers, and the consequent heavy workload^[21]^. Additionally, medical students interested in neurosurgery are required to seek training in developed countries due to the lack of neurosurgical training observed in developing countries^[21]^. Another issue involves the access to safe, efficient, and affordable surgical and anesthetic care^[21,22]^. Facilities providing such care are inaccessible for most populations because of the limited transportation to neurosurgical centers^[18]^. Individuals warranting acute care may need to utilize expensive private transportation because ambulances are frequently absent or hardly present in some locations^[23]^ (see Table 2).

**Table 2:** Challenges faced by LMICs in delivering neurosurgical care.

Technologies and developments	Financial support	Human resources	References
Lack of surgical materials	Expensive transportation	Lack of trained neurosurgeons	^[14,24]^
Lack of anesthesia	High cost of health services and surgeries	Uneven distribution of current limited doctors and nurses	^[25]^
Limited ambulances	Lack of insurance	Doctors require to travel to receive good training	^[26,27]^
Unsuitable ICU and operative rooms	Lack of funding	Heavy workload on healthcare providers	^[28,29]^
	Political influences	Inappropriate program structures and specific lecturers	^[27]^

### The role of neurosurgical nursing in LMICs

As a result of several challenges in the healthcare infrastructure in the LMICs, many studies were established to highlight the importance of neurosurgical nursing in these countries^[4,30,31]^. The scarcity in trained nurses result in numerous hurdles in terms of access to cost-effective neurosurgical facilities^[4]^. Contribution from LMICs and HICs showed good input in enhancing the healthcare sector while providing good solutions for daily and disaster periods^[30]^. Actually, these solutions must be supported by an enhancement in the nursing education, technology, telemedicine, and others; as well as an enrichment in supplementary resources, especially the workforce which includes nurses^[30]^. It is important to mention that nurses constitute half of the worldwide personnel in the healthcare sector, given that they partake in 90% of the interaction between the healthcare providers and the patients^[30]^. A study conducted in Indonesia and Pakistan – which constitute LMICs – observed that neurosurgery is a field that needs support of well-trained nurses to achieve successful outcomes^[31]^.

It is noteworthy to acknowledge the importance of holistic nursing care in addressing the patient’s physical, psychosocial, and spiritual needs^[32]^. It is evident that applying holistic care may show effective results in providing quality care, preventing disease, and reducing mortality rates, as stated by the World Health Organization (WHO)^[32,33]^. Many studies highlighted that this integrative method should be introduced by the nurses in their daily practice^[32-34]^. The degree of education, work environments, patient–nurse relationships, practice skills, management styles, and spirituality-promoting initiatives are all associated to holistic nursing care^[32,34]^.

**Figure 1.**
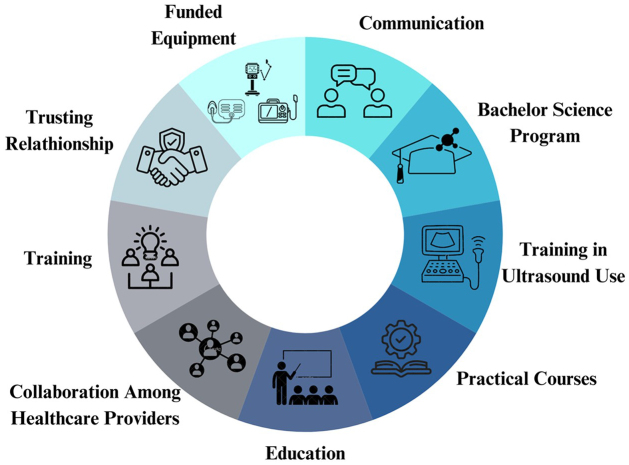
The collaborative approaches in neurosurgical nursing care.

Many approaches were implemented among healthcare providers and nurses to give the best healthcare quality to patients in the neurosurgical department. For example, the “twinning concept” focused on providing training to all the healthcare members, including nurses^[35]^ (Fig. 1). This approach was applied by professionals from HICs over 1 week in the aim of providing instructive and practical courses before, during, and after the surgical procedure to LMIC surgeons and nurses. Following this program, new funded equipment for the neurosurgical department was launched in LMICs in order to increase the capacity and corresponding training in its usage was provided to the nurses^[35]^. Moreover, many things were achievable due to the effort of nurses in cleaning and re-sterilizing the equipment^[35]^. In addition, intraoperative ultrasonography (USG) is more widely accessible in LMICs than computed tomography and magnetic resonance imaging^[36,37]^. For better usage of intraoperative USG, nurses are well-trained and equipped by professional ultrasonographers^[37]^. In Pakistan and Indonesia, four bachelor of science degrees were introduced to the nursing program in 2015 in order to improve the formation^[31]^.

Furthermore, several studies insisted on the importance of adopting dynamic collaboration among healthcare professionals to provide a patient-centered care^[35,38,39]^. For example, the National Surgical, Obstetrics, and Anesthesia Plans called for a collaboration among surgical nurses, radiologists, and reconstructive surgeons since neurosurgical care relies on a multidisciplinary approach^[39]^. Communication is essential to ensure patients with focal neurological symptoms – such as difficulties in hearing, speech, and perception – are not excluded from medical care^[40,41]^. Communication provides nurses with an opportunity to build rapport with their patients and respective relatives, enhancing the quality of care^[40]^ (see Fig. 1).

### Challenges and opportunities around neurosurgical nursing in LMICs

Neurosurgical nursing is a specialty that has been underrepresented even in developed countries. In LMICs, the shortage of surgical staff is compounded by a lack of well-trained nursing personnel responsible for the administration of neurosurgical wards. Many nurses lack skills necessary to fulfill their roles, as nurse practitioners not subspecialized in the field of neurosurgery are assigned the responsibility owing to a scarcity of both trained nurses and training programs meeting satisfactory standards^[29]^. In addition, most nurse practitioners and healthcare workers are forced to leave their countries for financial reasons, limited opportunities, poor working conditions, and interprofessional unresolved conflicts with clinicians, surgeons, or incompatible trained nurses alongside political issues and management corruption^[28]^. A stratagem to overcome these mitigating circumstances involves the improvement of nursing programs in developing countries and incorporating specific training centers that may graduate adequately trained neurosurgical nurses. Preparing top tier nursing care units for patients with neurological and trauma-related conditions necessitates specialized training. This education equips nurses with the ability to promptly identify, report, and respond to minor clinical and physiological changes, which can significantly impact patient prognoses^[42]^. Therefore, based on the specific clinical demands of these roles coupled with the broader needs of global neurotrauma care systems, new initiatives have been settled. These include novel approaches to interprofessional training and the promotion of nursing leadership to mitigate these issues effectively^[43]^. The systemic assessment of such initiatives would follow various tactics such as the regular evaluation of nursing workforce and the accessibility and caliber of accredited nursing programs which will allow the enhancement and development of surgical policies^[44]^. Also, establishing twinning programs with developed countries and those of high income is an essential asset in building and incorporating neurosurgical nursing in LMICs^[45]^.

### Advocacy and policy implications

Developing a sufficient infrastructure as well as providing necessary equipment ensures the delivery of an optimum health care system that offers all types of medical services including neurosurgical care. Neurosurgery is a field that unfortunately does not possess vast numbers of training professionals because of its necessity to utilize state-of-the-art equipment required for adequate clinical exposure of surgeons^[46]^. Therefore, international organizations should push forward to partner with companies who design affordable, durable, and good quality materials to be distributed among LMICs. Moreover, ensuring yearly attention and follow-up on these materials help in guaranteeing proper functioning in delivery of the best patient-centered care^[46]^. Furthermore, a highly neglected area in neurosurgery is undergoing and implementing research studies since it is considered to be an important asset in enhancing neurosurgery services^[46]^. So, collaboration of international wavers in funding research programs in LMICs is necessary^[46]^. Finally, neurosurgical professionals in LMIC should advocate for establishing training programs that promote education and improvement in the field to level up to training programs based in developed countries^[46]^.

## Conclusion

Competency, commitment, leadership, and knowledge are the main characteristics that result in a successful career for neurosurgical nurses. Nurses have many responsibilities in the pre-operative period to ensure the patient’s eligibility for the surgical procedure, as well as in the post-operative phase to provide the best quality care and meet the patient’s needs. However, in LMICs, there is a shortage of healthcare services, including technologies, innovations, and funding, alongside human resources, which limits nursing care for neurosurgical patients. Thus, HICs and LMICs should establish further programs that allow dynamic collaboration between countries to enhance the quality of care for neurological patients. LMICs should also enact additional policies to facilitate the implementation of training programs, the improvement of nursing school curricula, and the use of foreign expenditure. Moreover, further research should be conducted in LMICs to bolster the importance of neurosurgical nursing care in these countries. It would be fundamental to investigate the efficacy of telemedicine for neurosurgical consultations in low- and middle-income countries, alongside neurosurgical nurses providing patients with the information they require for impending surgery.

## Data Availability

Not applicable.
